# Parametric Optimization for Quality of Electric Discharge Machined Profile by Using Multi-Shape Electrode

**DOI:** 10.3390/ma15062205

**Published:** 2022-03-16

**Authors:** Fouzia Gillani, Taiba Zahid, Sameena Bibi, Rana Sami Ullah Khan, Muhammad Raheel Bhutta, Usman Ghafoor

**Affiliations:** 1 Department of Mechanical Engineering and Technology, Government College University, Faisalabad 38000, Pakistan; fouziagillani@gcuf.edu.pk; 2 Department of Mechanical Engineering, Institute of Space Technology, Islamabad 44000, Pakistan; 3 NUST Business School, National University of Sciences and Technology (NUST), Islamabad 44000, Pakistan; taibazahid_35@yahoo.com; 4 Department of Mathematics, Air University, Islamabad 44000, Pakistan; sameenabibi@mail.au.edu.pk; 5 Department of Mechatronics and Biomedical Engineering, Air University, Islamabad 44000, Pakistan; 211869@students.au.edu.pk; 6 Department of Computer Science and Engineering, Sejong University, Seoul 05006, Korea

**Keywords:** EDM, multi-shape electrode, ANOVA, regression analysis

## Abstract

The electrical discharge machining (EDM) process is one of the most efficient non-conventional precise material removal processes. It is a smart process used to intricately shape hard metals by creating spark erosion in electroconductive materials. Sparking occurs in the gap between the tool and workpiece. This erosion removes the material from the workpiece by melting and vaporizing the metal in the presence of dielectric fluid. In recent years, EDM has evolved widely on the basis of its electrical and non-electrical parameters. Recent research has sought to investigate the optimal machining parameters for EDM in order to make intricate shapes with greater accuracy and better finishes. Every method employed in the EDM process has intended to enhance the capability of machining performance by adopting better working conditions and developing techniques to machine new materials with more refinement. This new research aims to optimize EDM’s electrical parameters on the basis of multi-shaped electrodes in order to obtain a good surface finish and high dimensional accuracy and to improve the post-machining hardness in order to improve the overall quality of the machined profile. The optimization of electrical parameters, i.e., spark voltage, current, pulse-on time and depth of cut, has been achieved by conducting the experimentation on die steel D2 with a specifically designed multi-shaped copper electrode. An experimental design is generated using a statistical tool, and actual machining is performed to observe the surface roughness, variations on the surface hardness and dimensional stability. A full factorial design of experiment (DOE) approach has been followed (as there are more than two process parameters) to prepare the samples via EDM. Regression analysis and analysis of variance (ANOVA) for the interpretation and optimization of results has been carried out using Minitab as a statistical tool. The validation of experimental findings with statistical ones confirms the significance of each operating parameter on the output parameters. Hence, the most optimized relationships were found and presented in the current study.

## 1. Introduction

**Figure 1:**
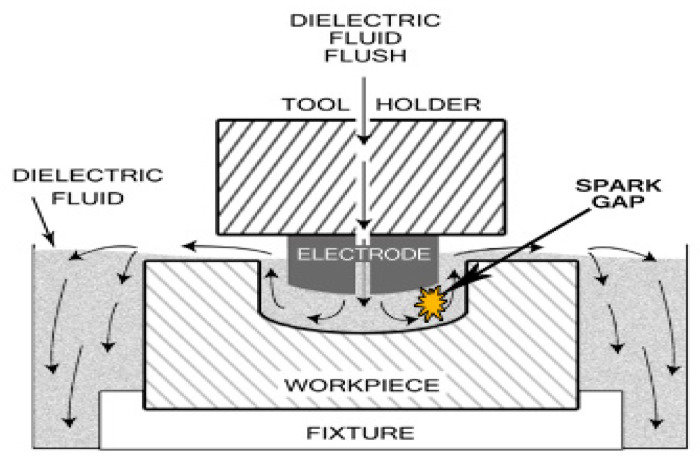
Die sinker EDM.

Modern manufacturing industries are facing challenges from advanced materials, viz., super alloys, composites and ceramics, that are difficult to machine and which require better surface quality and high accuracy, which increases overall machining cost. To overcome these challenges, non-traditional methods of machining are being employed to achieve a better surface finish, a higher metal removal rate and greater dimensional accuracy with less tool wear [1]. Electrical discharge machining (EDM), an electrothermal, non-traditional material removal process, which is widely used to produce parts such as punches, dies, molds and finished parts for automobiles and the aerospace industry [2]. The above technique is not new, as it was developed in the late 1940s [3]. The working principal of electric discharge machining (EDM) is based upon the electrical spark generated between anode and cathode, and extreme heat is produced near to the melt zone and, as a result, materials are dissipated [4]. The workpiece and the apparatus are submerged in a dielectric liquid [3]. A known designed gap, called a spark gap, is maintained between the tool and the workpiece surfaces. There is no direct contact between the electrode and the work piece; therefore, mechanical stresses, chatter and vibration problems during machining are eliminated [4]. Materials of any hardness can be cut, as long as the material can conduct electricity [5]. EDM is a very demanding and complex process. The mechanism of the process is shown in Figure 1, though it is complicated and not entirely understood. Therefore, it is difficult to develop an optimal analytical model that can exactly predict the performance and optimal response by correlating the process parameters [6]. The EDM process involves a large number of input parameters, such as spark voltage, pulse-on time, pulse-off time, discharge current, duty cycle, dielectric flushing pressure, open circuit voltage, electrode material, electrode polarity, pulse wave form and frequency, inter-electrode gap and dielectric fluid [7]. Due to the distinctive property of machining using the thermal energy of difficult-to-machine and conductive materials, it is best suited to manufacturing surgical components [8,9]. For intricate shapes, the machining performance varies across the item being produced. The machining results for complex areas, such as sharp corners and pointed areas, is totally different because of different current density.

The quality of EDM-machined profiles is measured using various output parameters, such as surface roughness, wear ratio, hardness, etc. In an investigation conducted on mild steel to find the effect of the electrode shape on the material removal rate (MRR), electrode wear rate (EWR), wear ratio (WR) and average surface roughness (Ra), it was observed that round-shaped electrodes give the maximum of each value, followed by the square, triangular and the diamond-shaped electrodes. Diamond-shaped electrodes have the largest peripheral length compared to the other electrodes, which results in more heat loss to the surrounding area and finally causes low MRR [9,10]. A case study on quality and productivity optimization in EDM highlights the EDM of stainless steel, in which the best process environment has been determined to satisfy productivity and quality requirements simultaneously [11]. The EDM of D3 tool steel was carried out, presenting a mathematical model which depicts that there is not a single combination of levels that is optimal for a high MRR and better surface quality [12]. To investigate the effect of all process parameters on response, a proper DOE is required. The Taguchi method is a good tool in DOE to reduce noise in the system and the number of experimental runs. During the machining of a C–C composite, the Taguchi method has been implemented in the EDM process [13,14]. The optimization of HSS M2 grade and D2 steel has been achieved by implementing the DOE for the experimental runs [15,16]. The MRR influenced by peak current in AISI D3 material has been investigated [14]. Ni-Cr-Mo steel was machined to identify a relationship between material removal and tool wear ratio, showing that open voltage was the most significant factor in this relation [13]. A high MRR was achieved in the case of positive extremity, while good surface quality was achieved in negative extremity [17,18]. EDM with respect to economic aspects was discussed, keeping in view the input and output parameters [19]. Peak current has been found to be the most important influencing factor for surface roughness for EN 31 die steel [20,21]. An initial speedy rise in surface roughness (SR) with a rise in pulse-off time was found for an aluminium-7075 metal matrix, with the SR slowly decreasing as the pulse-off time rose further. Such droppage is due to the negative polarity of electrodes, which further yields uneven surfaces [22,23]. A connection between discharge current, pulse-on time and surface crack formation has been found while machining D2 and H13 tool steel with EDM [24]. The characteristics of machining of T6–Al7075 were identified using the spark gap, peak current and pulse-off time to record MRR, surface roughness and tool wear rate as output parameters. ANOVA was implemented to determine the significance factor from input parameters with relation to the output responses, and further optimization was achieved using TOPSIS [6,24,25,26]. In the case of EDM and its allied processes, optimization-related works were based on RSM. This optimization technique was used in the EDM process to maximize the MRR, although it reduced the TWR and EWR, improved the SR and SQ, keeping the cost at a lowest value [26,27]. EDM objectives are well defined, such as lower tool wear rate, better surface finish rates, lower power consumptions and high material removal rate. The latest optimization techniques used in EDM provide better-quality at lower costs and power consumptions [28]. Every method employed in the EDM process has the same targets: to enhance the capability of machining performance by adopting the better working conditions and to develop techniques to machine new materials [29,30]. The machining efficiency is largely dependent upon the selection of machining parameters. It is evident from the literature that peak current (Ip), pulse-on time (Ton), duty cycle (TAU) and voltage gap (V) were found to have significant influence on surface roughness (SR) [31]. A detailed review of the literature revealed that although machining inaccuracies can be reduced through the optimization of input parameters, which actually improves the machining rate and the machining accuracy of EDM, this current work aims to investigate the potential procedure parameters affecting the dimensional accuracy, surface roughness and hardness when machining D2 die steel using the electric discharge machining process. This paper explores the connection between spark voltage (Vo), pulse-on time (Ton), depth of cut (DOC) and current (A) on surface roughness (SR), dimensional accuracy and hardness of the profile. A full factorial design of experiment was implemented to carry out the experiments on the EDM of a die sinker. Analysis of variance (ANOVA) and signal-to-noise ratio (S/N ratio) were implemented to find out the optimum independent parameters and their levels, as three level values have been used for each input parameter. The regression analysis is used to analyze the relationship between dependent and independent parameters. Previously, the optimization of process parameters for EDM was analyzed with less experimental exertion than the current work, which actually led to more than two hundred experiments using a full factorial design of experiment approach. Additionally, a novel profile was selected which includes intricate shapes, such as inner and outer curvatures, along with three angles, 30°, 60° and 90°, which actually considers the major complexity of the profiles while machining via EDM. The effectiveness of the EDM process with a copper electrode is evaluated in terms of the surface roughness, hardness and accuracy of the machined profile. The evaluation of the quality of the die steel profile includes assessing the effect of current, pulse-on time, spark voltage and depth of cut on the accuracy, surface roughness and hardness of the profile. The results obtained also contribute a lot to the existing data relating to the overall quality of the EDM when creating intricate profiles. Most of the optimized process parameter findings will give researchers the most appropriate level of values for process parameters while dealing with angles and curvatures.

## 2. Methodology

### 2.1. Experimental Setup

The chemical properties and mechanical composition of material D2 die steel can be observed in the literature [32], and are also shown in Table 1 and Table 2, respectively [33]. The work piece used in this experiment is D2 die steel (25.4 mm diameter and 6 mm thickness). Then, with the help of an EDM die sinker, the machining operation was performed. Voltage fluctuation, machine off-set, dielectric fluid and a copper electrode with a diameter of 25.4 mm were the fixed process parameters. A specifically designed multi-shaped copper electrode was used as a tool. The tool was designed to include five geometrical shapes in its outer profile. Table 3 shows the control factors and their corresponding levels. The mechanical equipment, namely, a surface texture meter with at least a count of 0.006 µm by Taylor Hobson, Leicester UK, a Rockwell Hardness Tester with 60 KN of force by Industrial Physics, UK and a coordinate measuring machine (CMM) with an accuracy of up to 1 micron by Eley Metrology, Derby, UK were used to measure the surface roughness, hardness and accuracy of the machined profile, respectively.

### 2.2. Tool Shape

**Figure 2:**
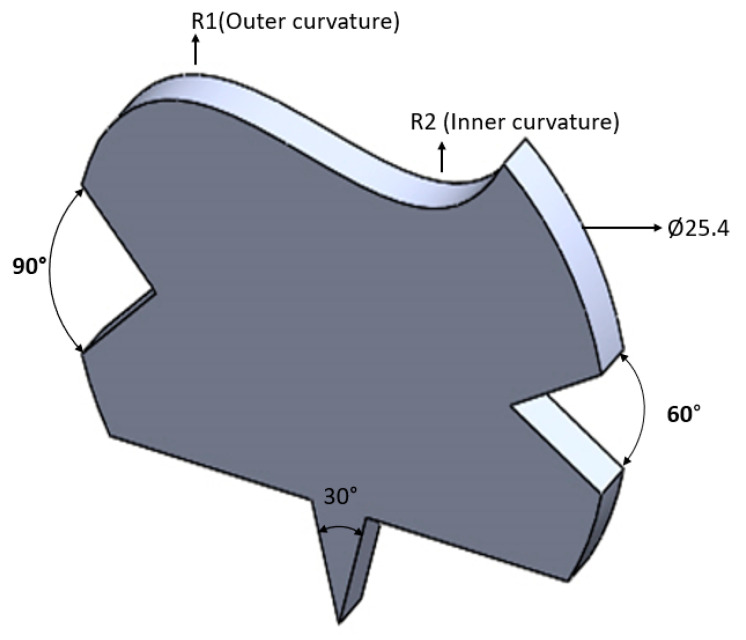
Tool geometry.

The tool was made of copper and designed as shown below in Figure 2.

### 2.3. Fixed EDM Conditions

The EDM die sinker CJ-230 used for the experimentation has the fixed conditions and specifications outlined in Table 4.

## 3. Experiments and Results

The experimental design was carried out using a full factorial design of experiment (DOE) approach, which utilizes a large number of experimental runs, with nearly 243 experiments performed in this study. There were more than two input parameters; therefore, the above-mentioned DOE was adopted. The design of experiment approach helps to generate the performance characteristics data that are close to the ideal ones, rather than any other data within a specified range, thereby improving the overall quality of the product [34]. This said design of experiment was implemented and employed with the statistical tool called ANOVA (analysis of variance) in order to determine the significance and percentage contribution of individual process parameters on the performance characteristics or output parameters. The effect of machining parameters in electric discharge machining on the characteristics of a D2 die steel workpiece has been investigated. The quality of the machined profile, determined on the basis of surface roughness, accuracy of the angles and surfaces and hardness of the profile, has been considered as responses, while the machining variables are pulse-on time (T_ON_), depth of cut (DOC), current (I_p_) and spark voltage (V_p_). The above selected process parameters were selected by extensively examining the trends in the existing literature in the selected area. This helped us find the most optimized and significant process parameters covering most of the intricate shapes to be machined via EDM. The above-mentioned parameters have also been measured using, a surface texture meter with at least a count of 0.006 µm by Taylor Hobson, Leicester UK, a Rockwell Hardness Tester with 60 KN of force by Industrial Physics, UK and CMM with an accuracy of up to 1 micron by Eley Metrology, Derby, UK respectively. The significant factors that critically effect the machining characteristics are observed and discussed in the following section.

## 4. Data Analysis for Accuracy of Profile

In this section, the data regarding the accuracy of the profile, including angles and radius, obtained from the experiments are analyzed. As described earlier, these experiments were carried out by varying the process parameters, using three level values for each (pulse-on time, depth of cut, current and spark voltage) in the EDM of D2 die steel. As mentioned above, the accuracy of the profile at different angles and curvatures has been theoretically measured using a coordinate measuring machine, which measures the deviation of the actual machined profile from the designed profile by calculating the difference between the primary and secondary plane taken for the plate and eroded profile, respectively. The accuracy of CMM was taken up to 1 micron. The analysis of variance ANOVA for the accuracy of the profile analyzed data from 243 experiments using an EDM die sinker. The F factor was calculated for a 95% level of confidence. The values which are less than 0.05 are not significant and the model is sufficient to represent the relation between the machining parameters and responses. The results were analyzed using ANOVA in Minitab for finding out the significant factors affecting the performance measures. Later on, the results of the ANOVA were verified using regression models. The principle for the F-test is that the larger the F value for a specific parameter, the greater the effect on the performance characteristics would be due to a change in that process parameter. The ANOVA table shows that different combinations of pulse-on time, depth of cut, current and spark voltage significantly affect the accuracy of the profile at different angles (30°, 60° and 90°). Also Figure S1 in shows optical measurements of angles (30°, 60° and 90) and curvatures via CMM with an accuracy of up to 1 micron by Eley Metrology, Derby, UK.

## 5. Main Effect Plots

**Figure 3:**
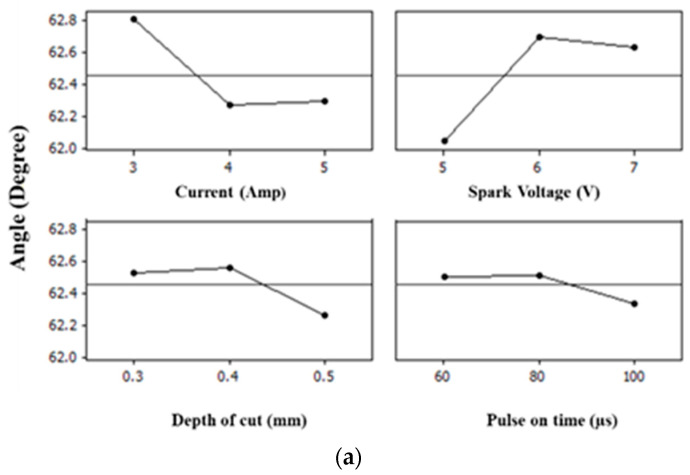

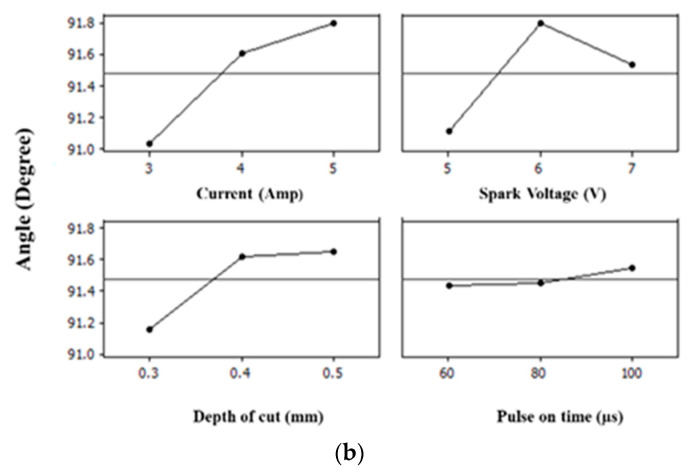
Main effect plots for accuracy of profile at different angles. Part (**a**) of Figure 3 shows the effect of all four input parameters on the accuracy at an angle of 60°, whereas (**b**) shows the effects of same parameters at an angle of 90°.

**Figure 4:**
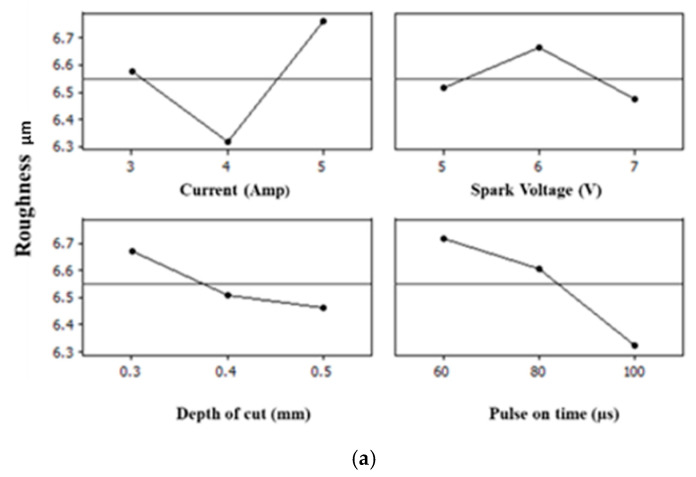

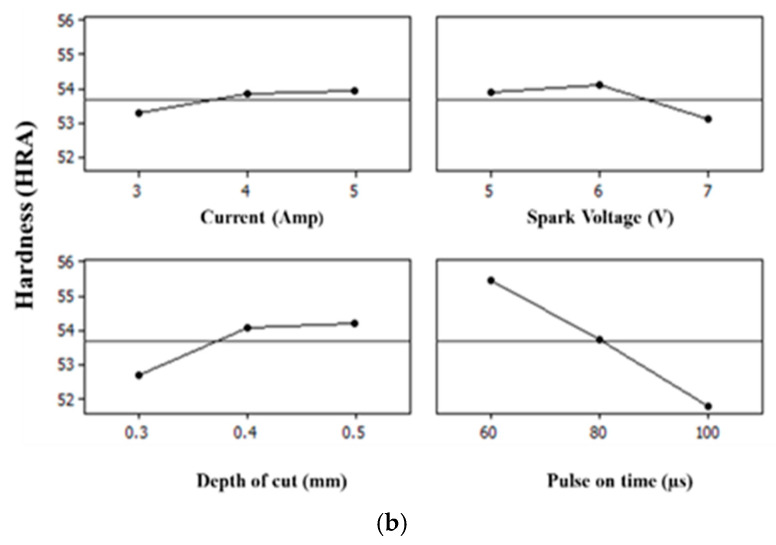
Main effect plots for surface roughness and hardness of the machined profile. Here in above Figure 4, (**a**) shows the effect of all four input parameters on the surface roughness (amplitude parameter), and (**b**) presents the effects of same parameters on the hardness of the machined profile.

A main effect plot is a graph that shows the average or means of response at each level of the factor or input parameter. The main effect plot helps to determine the influence of individual input parameters on the responses being measured [3]. The main effect plots of each response are shown in Figure 3a,b and Figure 4a,b and explained below.

## 6. Discussions

In this study, four process parameters, namely, spark voltage (V), current (I_P_), pulse-on time (T_on_) and depth of cut (DOC), were employed to investigate their impact on the quality parameters in the current study, i.e., the accuracy of the profile, surface roughness and hardness of the EDM-machined profile of D2 die steel. It was expected that the process parameters would have both individual and combined effects on the output parameters mentioned above. The results obtained support the expectation: current (I_P_) and spark voltage (V) both inversely affect the accuracy of angles in the range of 3 A to 4 A and 5 V to 6 V, respectively. After this, the values of the angles fall close to their actual value with the current (I_P_) in the range of 4 A to 5 A and, for spark voltage, in the range of 6 V to 7 V. Similar behavior is observed in the case of the accuracy of the inner and outer curvatures. The hardness of the profile is directly increased from 53 HRA to 54 HRA in the current (I_P_) range of 3 A to 4 A and the spark voltage (V) range of 5 V to 6 V; further, there is a slight increase in the hardness during the current (I_P_) range of 4 A to 5 A due to an increase in the carbon deposition on the surface of the profile. Similar behavior and reasons have also been found for the machined surfaces of solutions treated and aged with Inconel 718 [35]. However, hardness was observed to change inversely to the spark voltage range of 6 V to 7 V. Whereas, statistically, it is observed that surface roughness is strongly affected by the change is current (I_P_), the roughness of the profile decreases from 6.6 to 6.3 in the current (I_P_) range of 3 A to 4 A and increases slightly with the increase in the spark voltage (V) from 5 V to 6 V, while it sharply increases from 6.3 to 6.7 in the current (I_P_) range of 4 A to 5 A. This is quite logical because an increase in the peak current produces a strong spark, which generates the higher temperature, causing more material to melt and erode from the work piece, creating a rough surface. It is reported in the literature that the surface roughness for a graphite electrode increases with the increase in the pulse-on time until a certain value (peak), whereas for copper and copper–tungsten electrodes, the surface roughness decreases as the pulse-on time increases. Therefore, the diverse effect of the pulse-off time on surface roughness is apparent [36]. In the present research, the surface roughness decreased from 6.6 to 6.4 when increasing the spark voltage (V) from 6 V to 7 V.

The depth of cut (DOC) inversely affects the accuracy of the profile at the tested angles and curvatures in its range of 0.3 mm to 0.4 mm, whereas it is directly proportional to the accuracy of angles and profiles in its range of 0.4 mm to 0.5 mm. The surface roughness is inversely affected by the depth of cut, and it is observed that with the increase in the depth of cut, surface roughness increased, which is also logical. The depth of cut of the operation is proven to be an important factor that affects the surface hardness, which means that the deeper the profile (DOC is higher), the longer the operation time (pulse-on time) where the surface is exposed to the heat released by the spark energy [37]. In the case of hardness, it was expected that a heat treatment process involving continuous heating and quenching at the surface would harden the surface layer or recast layer, which means that hardness would be increased if the machining time were high, along with the DOC [38]. The results of the current study fully support these expectations, as hardness increased from 52.5 HRA to 54 HRA in depth of cut (DOC) range from 0.3 to 0.4 and slightly increased with the increase in the depth of cut (DOC) from 0.4 mm to 0.5 mm. Similarly, by increasing the pulse-on time (T_ON_), the accuracy of the profile at the angles decreased and increased on the curvatures in its range of 60–80 µs. Further, it was increased at the angles and decreased at the curvatures in the range of 80 µs to 100 µs, whereas the roughness of the profile decreased from 6.7 to 6.6 in the range of 60–80 µs and decreased further when the pulse-on time (T_ON_) increased from 80 µs to 100 µs. The hardness of the profile is inversely proportional to the pulse-on time (T_ON_) in the range of 60–80 µs and 80 µs to 100 µs. Therefore, the obtained results clearly show that DOC and current (I_p_) are more significant individually, whereas their combined effects with each other and along with the spark voltage (V) and pulse-on time (T_ON_) mean they less significantly affect the output parameters. While performing the experimentation with an EDM die sinker and an instrumentation-based analysis, few errors and limitations contributed towards the final results, as in the analysis is confirmed by the ANOVA tables (presented in supplementary materials) from Tables S1–S7 for each response variable after performing the statistical analysis. However, some limitations and expected errors must be considered when drawing conclusions from this research. During instrumentational analysis, human error while placing the samples must be considered in the current work. Additionally, the EDM die sinker must be placed at STP conditions. As intense heating and cooling happened while machining, the tool may deuterate. If a worn-out tool surface is used for sample cutting, this also leads to inaccurate results. Flushing pressure during machining with EDM is also an important factor as it contributes towards the accuracy of the profile, as well as the wear rate of the tool, along with the hardness of machined surface.

### Effects of Process Parameters on Performance Measures

From the above main effect plots for the performance, measured using an ANOVA and counter verified by regression analysis, it can be concluded that when the angle was at 30°, the accuracy of the inner and outer curvatures did not show significant deviation for all four inputs, whereas at 60° and 90°, the current, spark voltage and depth of cut showed significant deviation from the mean values. However, in the case of the surface roughness of the machined profile, current and pulse-on time had significant values for surface roughness, whereas the combined effect of current and pulse-on time was also found to be significant. The interaction plot was also plotted, where each plot exhibits the interaction between four different machining parameters, namely, current (I_p_), pulse-on time (T_ON)_, depth of cut (DOC) and spark voltage (V). This implies that the effect of one factor is dependent upon another factor. The hardness measured at 60 KN was affected due to the depth of cut and pulse-on time, probably because of the flow of heavy current resulting in the deposition of carbon on the surface of the machined profile. This deposition is due to the rapid cooling effect during pulse-off time.

## 7. Conclusions

In this study, the experiment was conducted by considering four variable parameters, namely, current, pulse-on time, depth of cut and spark voltage. The objective was to conduct the parametric optimization by finding out the effects of the variable parameters on the quality of the machined profile. The tool material was taken as copper and the workpiece was chosen as AISI D2 die steel. Using the full factorial method, a design of experiment approach was developed to perform the experiments using three level values for each input. The following conclusions were drawn: (i) the accuracy of the angles at higher values is significantly affected under the selected input parameters, whereas the accuracy of curvatures is not changed significantly, (ii) the roughness of the machined profile is significantly enhanced due to heavy current and higher spark voltage, (iii) the hardness of the machined profile was enhanced due to rapid cooling during the off time of the operation and probably due to heavy current flow and (iv) the present study is extremely useful for improving the overall quality of the machined profile.

Future study on this method may investigate the following aspects. In this study, only four machining parameters were selected. A detailed study may be performed considering other parameters also, such as servo voltage and flushing pressure. Instead of a copper electrode, other electrode material, such as graphite brass, may be used. Different grades of tool steels can be used for the investigation of machining parameters for the interest of industries. There are several optimization tools, viz., RSM, ANN, ANFIS, GA and WPCA, that may be employed for the parametric optimization procedure.

## Figures and Tables

**Table 1:** Specifications of EDM die sinker.

Fixed Specifications	Values
Incoming voltage	(220/380) V
Maximum average output	(30–200) A
No load output	100 µs
On-time setting range	(1–9999) µs
Off-time setting range	(1–9999) µs
Electro-spark frequency	500 Hz–500 KHz
Dielectric used	Kerosene

**Table 2:** Chemical composition of D2 die steel [32].

Material	% Composition
C	1.4–1.6
Mn	0.6
Si	0.6
Co	1
Cr	11–13
Mo	0.7–1.20
V	1.10
P	0.03
Ni	0.30
Cu	0.25
S	0.30

**Table 3:** Mechanical properties of D2 die steel [32].

Mechanical Properties	Metric	Imperial
Hardness, Rockwell C	62	62
Hardness, Vickers	748	748
Izod impact unnotched	70.0 J	56.8
Poisson’s ratio	0.27–0.30	0.27–0.30
Elastic modulus Ksi	190–210 GPa	27,557–30,457

**Table 4:** Control factors and their level values.

Process Parameter	Symbol	Unit	Level-1	Level-2	Level-3
Spark voltage	V_p_	Volt	5	6	7
Pulse-on time	T_ON_	µ-s	60	80	100
Depth of cut	DOC	mm	0.3	0.4	0.5
Current	I_p_	Amp	3	4	5

There are three level values for each control factor.

## Data Availability

The prepared samples to support the findings of this study can be made available from the corresponding authors upon reasonable request.

## Supplementary Materials

The following are available online at https://www.mdpi.com/article/10.3390/ma15062205/s1, Table S1: ANOVA for Accuracy of angles 30°; Table S2: ANOVA for Accuracy of angles 60°; Table S3: ANOVA for Accuracy of angles 90°; Table S4: ANOVA for Accuracy of Inner Curvature; Table S5: ANOVA for Accuracy of Outer Curvature; Table S6: ANOVA for Surface Roughness; Table S7: ANOVA for Hardness; Figure S1: CMM images of the machined profile at angles and curvatures for the observance of Accuracy.

## Abbreviations

ANOVA	Analysis of Variance
I_P_	Current
DOE	Design of Experiments
EDM	Electric Discharge Machining
T_ON_	Pulse-on Time
DOC	Depth of Cut
MRR	Material Removal Rate
V_P_	Spark Voltage
