# Supplementary material for: Parametric Optimization for Quality of Electric Discharge Machined Profile by Using Multi-Shape Electrode
Source: Materials (Basel). 2022 Mar 16;15(6):2205. doi: 10.3390/ma15062205 (PMC8955625; doi:10.3390/ma15062205)
Supplement: Supplementary file 1 [file materials-15-02205-s001.zip › materials-1604055-supplementary.pdf]

Table S1. ANOVA for Accuracy of angles 30º

Source	DOF	Seq SS	Adj SS	Adj MS	F	P%
Current (I_P)	2	5.909	5.909	2.954	0.64	0.530
Spark Voltage (V_P)	2	17.225	17.225	8.613	1.86	0.049
Depth of Cut (DOC)	2	1.366	1.366	0.683	0.15	0.863
Pulse on time (T_{ON})	2	9.425	9.425	4.713	1.02	0.044
Current × spark voltage	4	4.479	4.479	1.120	0.24	0.914
Current × Depth of cut	4	18.781	18.781	4.695	1.01	0.403
Current × pulse on time	4	5.878	5.878	1.469	0.32	0.866
Spark voltage × depth of cut	4	18.316	18.316	4.579	0.99	0.416
Spark voltage × pulse on time	4	15.092	15.092	3.773	0.81	0.518
Depth of cut× pulse on time	4	20.451	20.451	5.113	1.10	0.357
Current ×spark voltage ×depth of cut	8	60.926	60.926	7.616	1.64	0.117
Current ×spark voltage × pulse on time	8	26.746	26.746	3.343	0.72	0.673
Current × depth of cut × pulse on time	8	27.458	27.458	3.432	0.74	0.656
spark voltage × depth of cut × pulse on time	8	38.783	38.783	4.848	1.05	0.404
Current ×spark voltage × depth of cut × pulse on time	16	21.767	21.767	1.360	0.29	0.997
Error	162	751.286	751.286	4.638		
Total	242	1043.889	1043.889			7.807

Seq, SS, sum of squares;
DOF, Degree of freedom;
Adj MS, Adjusted Mean square
P, Percentage contribution;
Significance at 95% confidence level.

Table S2. ANOVA for Accuracy of angles 60º

Source	DOF	Seq SS	Adj SS	Adj MS	F	P%
Current (I_P)	2	15.132	15.132	7.566	2.08	0.128
Spark Voltage (V_P)	2	20.894	20.894	10447	2.88	0.059
Depth of Cut (DOC)	2	4.191	4.191	2.095	0.58	0.563
Pulse on time (T_{ON})	2	1.494	1.494	0.747	0.21	0.814
Current × spark voltage	4	63.831	63.831	15.958	4.40	0.002
Current × Depth of cut	4	20.361	20.361	5.090	1.40	0.236
Current × pulse on time	4	28.061	28.061	7.015	1.93	0.107
Spark voltage × depth of cut	4	18.797	18.797	4.699	1.29	0.274
Spark voltage × pulse on time	4	7.318	7.318	1.830	0.50	0.733
Depth of cut× pulse on time	4	3.565	3.565	0.891	0.25	0.912
Current ×spark voltage ×depth of cut	8	39.794	39.794	4.974	1.37	0.213
Current ×spark voltage × pulse on time	8	15.603	15.603	1.950	0.54	0.827
Current × depth of cut × pulse on time	8	22.928	22.928	2.866	0.79	0.612
spark voltage × depth of cut × pulse on time	8	17.481	17.481	2.185	0.60	0.775

Current ×spark voltage × depth of cut × pulse on time	16	86.335	86.335	5.396	1.49	0.110
Error	162	588.017	588.017	3.630		
Total	242	953.801				6.425

Table S3. ANOVA for Accuracy of angles 90º

Source	DOF	Seq SS	Adj SS	Adj MS	F	P%
Current (I_P)	2	28.859	28.859	12.930	2.58	0.079
Spark Voltage (V_P)	2	19.310	19.310	9.655	1.92	0.149
Depth of Cut (DOC)	2	12.331	12.331	6.166	1.23	0.295
Pulse on time (T_{ON})	2	0.573	0.573	0.287	0.06	0.944
Current × spark voltage	4	86.621	86.621	21.655	4.32	0.002
Current × Depth of cut	4	26.649	26.649	6.662	1.33	0.262
Current × pulse on time	4	31.430	31.430	7.858	1.57	0.186
Spark voltage × depth of cut	4	33.231	33.231	8.308	1.66	0.163
Spark voltage × pulse on time	4	10.483	10.483	2.621	0.52	0.719
Depth of cut× pulse on time	4	26.767	26.767	6.692	1.33	0.260
Current ×spark voltage ×depth of cut	8	156.023	156.023	19.503	3.89	0.000
Current ×spark voltage × pulse on time	8	37.843	37.843	4.730	0.94	0.483
Current × depth of cut × pulse on time	8	18.752	18.752	2.344	0.47	0.878
spark voltage × depth of cut × pulse on time	8	20.013	20.013	2.502	0.50	0.856

Current ×spark voltage × depth of cut × pulse on time	16	56.359	56.359	3.522	0.70	0.789
Error	162	812.566	812.566	5.016		
Total	242	1374.810				6.065

Table S4. ANOVA for Accuracy of Inner Curvature.

Source	DOF	Seq SS	Adj SS	Adj MS	F	P%
Current (I_P)	2	0.33386	0.33386	0.6693	3.23	0.049
Spark Voltage (V_P)	2	0.05223	0.05223	0.02611	0.51	0.604
Depth of Cut (DOC)	2	0.04038	0.04038	0.02019	0.39	0.677
Pulse on time (T_{ON})	2	0.34219	0.34219	0.17110	3.31	0.045
Current × spark voltage	4	0.08625	0.08625	0.02156	0.42	0.796
Current × Depth of cut	4	0.015057	0.015057	0.00264	0.05	0.995
Current × pulse on time	4	0.18868	0.18868	0.04717	0.91	0.458
Spark voltage × depth of cut	4	0.09020	0.09020	0.02255	0.44	0.782
Spark voltage × pulse on time	4	0.21897	0.21897	0.05474	1.06	0.378
0.91Depth of cut× pulse on time	4	0.25265	0.25265	0.06316	1.22	0.303
Current ×spark voltage ×depth of cut	8	0.39395	0.39395	0.04924	0.95	0.475
Current ×spark voltage × pulse on time	8	0.26407	0.26407	0.03301	0.64	0.744
Current × depth of cut × pulse on time	8	0.44311	0.44311	0.0553	1.07	0.385
spark voltage × depth of cut × pulse on time	8	0.57352	0.57352	0.07169	1.39	0.205

Current ×spark voltage × depth of cut × pulse on time	16					0.388
Error	162					
Total	242					7.284

Table S5. ANOVA for Accuracy of Outer Curvature.

Source	DOF	Seq SS	Adj SS	Adj MS	F	P%
Current (I_P)	2	0.29265	0.29265	0.14632	2.14	0.021
Spark Voltage (V_P)	2	0.47427	0.47427	0.23714	3.46	0.044
Depth of Cut (DOC)	2	0.00761	0.00761	0.00381	0.06	0.946
Pulse on time (T_{ON})	2	0.08601	0.08601	0.04300	0.63	0.535
Current × spark voltage	4	0.02227	0.02227	0.00557	0.08	0.988
Current × Depth of cut	4	0.017037	0.017037	0.4259	0.62	0.648
Current × pulse on time	4	0.06182	0.06182	0.01545	0.23	0.924
Spark voltage × depth of cut	4	0.36861	0.36861	0.09215	1.35	0.256
Spark voltage × pulse on time	4	0.10844	0.10844	0.02711	0.40	0.812
0.91Depth of cut× pulse on time	4	0.16089	0.16089	0.04022	0.59	0.672
Current ×spark voltage ×depth of cut	8	0.39705	0.39705	0.04963	0.72	0.670
Current ×spark voltage × pulse on time	8	0.85845	0.85845	0.10656	1.56	0.142
Current × depth of cut × pulse on time	8	0.26592	0.26592	0.03324	0.49	0.865
spark voltage × depth of cut × pulse on time	8	0.23308	0.23308	0.02914	0.43	0.905

Current ×spark voltage × depth of cut × pulse on time	16	0.67061	0.67061	0.04191	0.61	0.871
Error	162	11.09873	11.09873	0.06851		
Total	242	15.27077				

Table S6. ANOVA for Surface Roughness.

Source	DOF	Seq SS	Adj SS	Adj MS	F	P%
Current (I_P)	2	8.1201	8.1201	4.0600	4.41	0.014
Spark Voltage (V_P)	2	1.6095	1.6095	0.8047	0.87	0.419
Depth of Cut (DOC)	2	1.9503	1.9503	0.9751	1.06	0.349
Pulse on time (T_{ON})	2	6.7161	6.7161	3.3580	3.65	0.028
Current × spark voltage	4	17.1065	17.1065	4.2766	4.65	0.001
Current × Depth of cut	4	9.4289	9.4289	2.3572	2.56	0.041
Current × pulse on time	4	1.3254	1.3254	0.3314	0.36	0.837
Spark voltage × depth of cut	4	2.3859	2.3859	0.5965	0.65	0.629
Spark voltage × pulse on time	4	0.1077	0.1077	0.0269	0.03	0.998
0.91Depth of cut× pulse on time	4	0.5137	0.5137	0.1284	0.14	0.967
Current ×spark voltage ×depth of cut	8	5.2555	5.2555	0.6569	0.71	0.679
Current ×spark voltage × pulse on time	8	0.7476	0.7476	0.0934	0.10	0.999
Current × depth of cut × pulse on time	8	1.4663	1.4663	0.1833	0.20	0.991
spark voltage × depth of cut × pulse on time	8	0.2114	0.2114	0.0264	0.03	1.000

Current ×spark voltage × depth of cut × pulse on time	16	0.8239	0.8239	0.0515	0.06	1.000
Error	162	149.1507	149.1507	0.9207		
Total	242	206.9195				

Table S7. ANOVA for Hardness.

Source	DOF	Seq SS	Adj SS	Adj MS	F	P%
Current (I_P)	2	21.845	21.845	10.922	1.17	0.314
Spark Voltage (V_P)	2	43.070	43.070	21.535	2.30	0.104
Depth of Cut (DOC)	2	115.515	115.515	57.758	6.17	0.003
Pulse on time (T_{ON})	2	546.351	546.351	273.176	29.16	0.000
Current × spark voltage	4	29.802	29.802	7.451	0.80	0.530
Current × Depth of cut	4	90.007	90.007	22.502	2.40	0.052
Current × pulse on time	4	2.959	2.959	0.740	0.08	0.989
Spark voltage × depth of cut	4	39.929	39.929	9.982	1.07	0.375
Spark voltage × pulse on time	4	7.777	7.777	1.944	0.21	0.934
0.91Depth of cut× pulse on time	4	20.842	20.842	5.211	0.56	0.695
Current ×spark voltage ×depth of cut	8	50.967	50.967	6.371	0.68	0.709
Current ×spark voltage × pulse on time	8	33.935	33.935	4.242	0.45	0.887
Current × depth of cut × pulse on time	8	15.778	15.778	1.972	0.21	0.989
spark voltage × depth of cut × pulse on time	8	11.910	11.910	1.489	0.16	0.996

Current ×spark voltage × depth of cut × pulse on time	16	23.582	23.582	1.474	0.16	1.000
Error	162	1517.673	1517.673	9.368		
Total	242	2571.940				

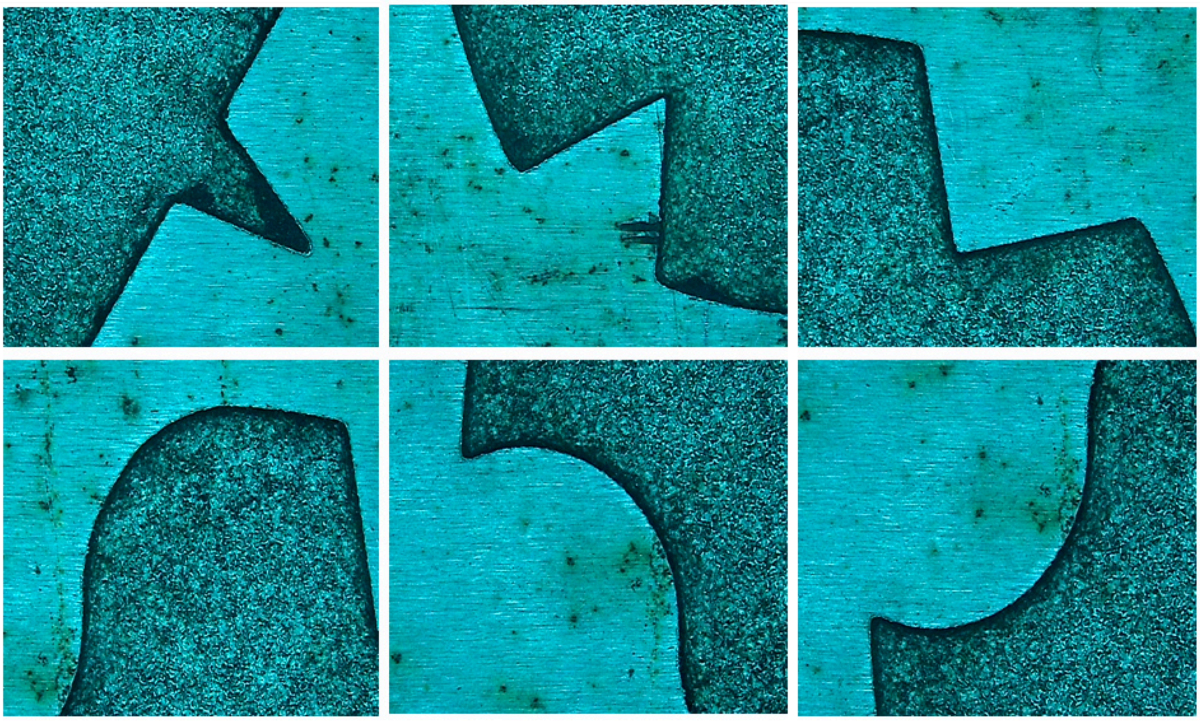

Figure S1. CMM images of the machined profile at angles and curvatures for the observance of Accuracy.
